# Outcome of femoral fractures treated with cerclages and intramedullary nailing

**DOI:** 10.1007/s00068-025-02883-x

**Published:** 2025-05-20

**Authors:** Franziska Ziegenhain, Claudio Canal, Sascha Halvachizadeh, Anne Mittlmeier, Hans-Christoph Pape, Christian Hierholzer, Valentin Neuhaus

**Affiliations:** 1 Division of Trauma Surgery, University Hospital Zurich (USZ), University of Zurich (UZH), Raemistrasse 100, 8091 Zuerich, Switzerland; 2 Department of Surgery, Spital Thurgau, Pfaffenholzstr. 4, 8501 Frauenfeld, Switzerland

**Keywords:** Femoral shaft fractures, Cerclage, Intramedullary nailing, Outcome

## Abstract

**Introduction:**

Femoral shaft fractures are a common entity in trauma surgery. The gold standard is closed reduction and intramedullary nailing. However, in some fracture patterns, the surgeon may choose to open the fracture for reduction. The purpose of this retrospective study was to analyze the outcome of femoral shaft fractures treated with open reduction, cerclage wiring and intramedullary nailing.

**Methods:**

We included adult patients with femoral shaft fractures treated with open reduction, cerclage wiring and intramedullary nailing at a level 1 trauma center in Switzerland during an 11-year period. The data collection was conducted retrospectively. Patient characteristics including age, sex and existing comorbidities were analyzed. Detailed information regarding the fracture patterns and surgical procedures was recorded. Outcome measures included the rates of malunion and nonunion, the time required for union, incidence of surgical site infections, and the frequency of revision surgeries.

**Results:**

We included a total of 69 patients, comprising 48 males and 21 females with a mean age of 50 years. A majority suffered a high velocity trauma (67%). The most common fracture type was multifragmentary subtrochanteric fracture. Approximately 57% of the patients underwent definitive surgical care within the first 24 h. Number of cerclages applied ranged from 1 to 4, with 18% positioned above the lesser trochanter. Delayed union occurred in 10% of cases, while nonunion was noted in 19% of patients.

Complications included femoral head necrosis in 3 (4%) patients, and surgical site infections were documented in 3 (4%) cases.

**Conclusion:**

Our findings give rise to closed reduction and internal fixation as a treatment of choice of femoral shaft fractures. We suggest the use of ORIF combined with cerclages, if closed reduction cannot be sufficiently achieved. However, a risk–benefit ratio should be assessed to minimize the risk of a higher complication rate with the use of ORIF.

## Introduction

Femoral shaft fractures represent a frequent injury and pose a common challenge for the trauma surgeon. The patterns of these fractures vary greatly. Elderly patients suffer from femoral shaft fractures through low-energy trauma, whereas in younger patients, these fractures are often caused by high-velocity trauma and are frequently accompanied by other serious injuries [1, 2]. Treatment options for femoral shaft fractures are, whenever possible, surgical, and include a variety of techniques like external fixation, plate or nail osteosynthesis, and in some rare circumstances primary arthroplasty. Closed reduction and intramedullary nailing is acknowledged as the gold standard [3]. The challenges of this treatment include correct patient positioning, achieving fracture reduction with correct length, axis and rotation and determining the appropriate entry point for the nail [4, 5]. The benefits of closed reduction and intramedullary nailing include the avoidance of extensive soft tissue damage, periosteal stripping and devascularization of bone fragments. However, in case of distinct fracture patterns, attaining these objectives can be challenging. To achieve adequate alignment and avoid complications such as malrotation, open reduction is a valid surgical strategy [6, 7]. To allow for intramedullary nailing, the surgeon needs to achieve reduction of the fracture elements before inserting the nail to achieve adequate results. An often discussed option is to perform open reduction and fracture stabilization using wire or cable cerclages [8, 9], which are designed to maintain the reduction and to maximize interfragmentary bone contact between fracture ends to improve bone healing [10]. Nonetheless, this technique requires a limited open approach and may significantly increase soft tissue damage including risk of serious damage to neurovascular structures during cerclage application and a higher risk of infections [11–13]. To understand which patients may benefit from open reduction and to identify risk factors for adverse outcomes, further research is essential. The aim of this study was to evaluate the characteristics of distinct femoral fractures treated with open reduction, cerclage wiring and intramedullary nailing.

## Material and methods

We conducted this study in strict accordance with relevant ethical guidelines, following approval from the Institutional Review Board of Kantonale Ethikkommission.

The research design of this study was a retrospective observational study with patients treated at a level I trauma center in Switzerland. Inclusion criteria were individuals aged 16 years and older who had sustained femoral fractures treated via open reduction, cerclage wiring and intramedullary nailing. We screened patients using the Swiss surgical classification “Schweizerische Operationsklassifikation (CHOP)” and the tenth edition of the International Classification of Diseases (ICD-10), codes and searching for key words in surgical notes [14, 15].

Patients with incomplete data sets or less than 12 months of follow-up were excluded.

Patient histories were thoroughly examined. Basic demographic information such as age and sex was recorded, along with details regarding the mechanism of trauma and injury pattern. Fractures were divided into three categories—pertrochanteric, subtrochanteric and femoral shaft fractures—and further categorized as simple or multifragmentary (3 or more fragments) based on the fracture pattern. Surgical procedures were analyzed for various aspects: timing of the surgery (definitive surgical treatment within 24 h, and if performed during day or night; we defined nighttime as operations performed between 8 PM and 7 AM.), types of implants used (including Gamma3 long nail (Stryker), Expert lateral femoral nail (Synthes) and T2 Femoral Nail (Stryker)), application and number of cerclages (above or below lesser trochanter), duration of surgery in minutes and fluoroscopy time in minutes, qualification of main surgeon (resident, junior attending, senior attending) and quality of reduction. The quality of reduction was evaluated based on modified Baumgaertner criteria and AO definition of on intra- and postoperative X-rays and was defined as good, acceptable or poor [16, 17]. To define outcome, we evaluated time to full weight bearing and fracture healing (time to union), surgical site infections, delayed union (3 to 6 months) or nonunion (more than 6 months), the necessity of revision surgery and the occurrence of femoral head necrosis.

### Statistics

Data for this study were processed using the Statistical Package for the Social Sciences (SPSS) software, version 29, (IBM Corp. Armonk, NY, USA). The findings are expressed as frequencies (numerical counts) and percentages, detailed in the tables provided. For particular variables, we reported ranges, covering follow-up, age, and duration of surgery.

The Chi-square test, Mann–Whitney U test, and Fisher's exact test were employed where appropriate to evaluate the association between the application of cerclages relative to the minor trochanter (above or below) and the incidence of femoral head necrosis, as well as to examine the relationships between various clinical factors and the occurrence of nonunion. These analyses involved comparative assessments across multiple variables, including the presence of open fractures (yes vs. no), injury mechanism (low-energy vs. high-energy), reduction quality (good vs. acceptable/poor), and the number of cerclages applied.

## Results

### Patient and fracture characteristics

We included 67 patients, accounting for a total of 69 femoral fractures. Of these, 48 patients (70%) were male with a mean age of 50 ± 21 years (ranging from 16 to 92 years). High-velocity trauma was the cause of injury in 67% (46 patients). Two patients (3%) suffered bilateral fractures. The fracture distribution was as follows: 5 (7%) trochanteric fractures, 28 (41%) subtrochanteric, 21 (31%) shaft and 15 (22%) multilevel fractures. Open fractures were reported in 6 (9%) of cases. Of these open fractures, two were classified as Gustilo type I, two cases as grade II and 2 cases as Gustilo IIIA [18]. Thirty nine patients (57%) underwent primary and definitive osteosynthesis within 24 h. The most commonly used implant was the Gamma3 long nail (Stryker) inserted in 51 (74%) of cases, followed by the expert lateral femoral nail (Synthes) in 11 (16%) of cases. Other intramedullary nails were used in 7 (10%) of the patients. (Table 1).

### Surgical procedures

Daytime surgeries accounted for 62% (43 cases) of the total. Junior attendings performed a majority of surgeries with 44 cases (64%), senior attendings conducting 14 cases (20%) and residents under supervision of an attending 11 cases (16%). The mean duration of surgery was 199 ± 77 min (ranging from 75 to 405 min). A subvastus approach was used in 57 cases (83%). The degree of reduction was good in 52 (75%), acceptable in 13 (19%), and poor in 4 (6%) of the cases.

Application of auxiliary cerclage stabilization was performed using 4 cerclages in 13% (9 cases), 3 cerclages in 22% (15 cases), 2 cerclages in 36% (25 cases), and a single cerclage in 29% (20 cases). Application of cerclages above the lesser trochanter level was limited to 18% (12 cases).

The mean fluoroscopy duration was 6.4 min (range 2 to 16.5 min). (Table 2).

### Outcome

The mean follow-up period was 36 ± 22 months with a range of 12 to 84 months. Uncomplicated osseous healing occurred in 71% (49 cases) of fractures. Delayed union, which ultimately resolved without revision, was observed in 10% (7 cases). Nonunion was reported in 13 (19%) cases with 5 (7%) cases being revised and ultimately healing, three (4%) revised but not healing yet, and one (1%) case pending revision. Three (4%) patients were lost to follow up after and one (1%) before revision. Femoral head necrosis occurred in three (4%) patients. Two out of three patients with femoral head necrosis had a cerclage above the lesser trochanter. All of those patients underwent an implant removal followed by a total hip replacement.

Surgical site infection after primary surgery in three (4%). Re-operation was needed in a total of 13 (19%) cases. The majority of these cases (*n* = 8, 12%) received re-osteosynthesis, followed by three (4%) prosthesis and two (3%) wound revisions. Hardware removal was performed in 21 (30%) of patients. One patient (1%) suffered surgical site infection (SSI) after hardware removal. (Table 3).

### Nonunion and femoral head necrosis

Our analysis revealed that none of the evaluated factors showed a statistically significant association with the occurrence of nonunion or femoral head necrosis. For nonunion, the presence of an open fracture (*p* = 0.076), injury velocity (low-energy vs. high-energy, *p* = 0.194), the quality of reduction (good vs. acceptable/poor, *p* = 0.283), and the number of cerclages used (*p* = 0.730) did not significantly influence the outcome. Additionally, the placement of cerclages above the lesser trochanter showed no significant association with femoral head necrosis, with a p-value of 0.076, where 2 out of 3 patients with necrosis had cerclages placed above the lesser trochanter.

## Discussion

The aim of our study was to evaluate the outcome of femoral shaft fractures, which were treated with open reduction, cerclage wiring and intramedullary nailing.

**Fig. 1:**
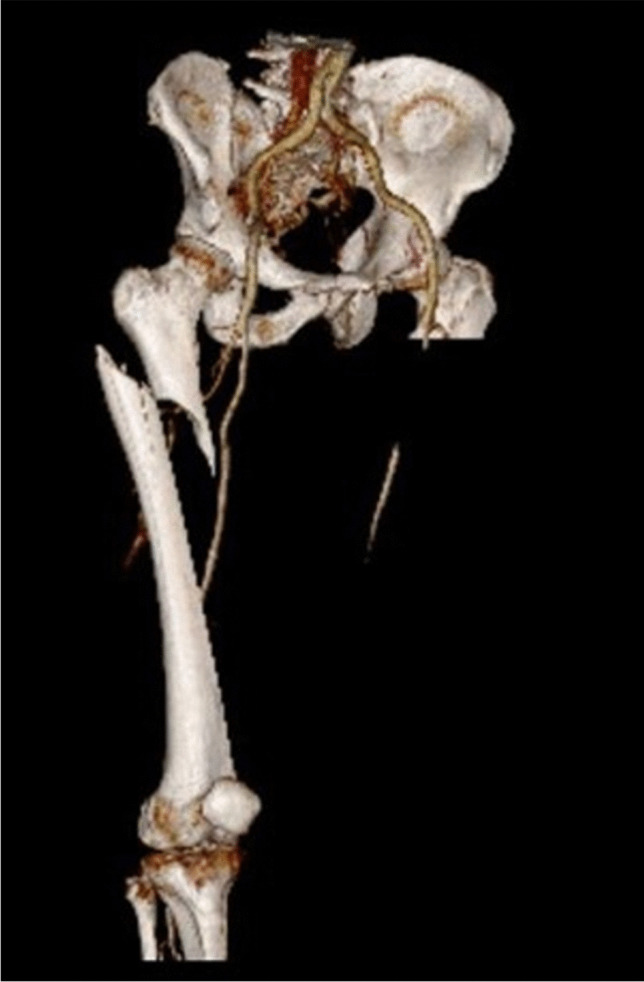
A 72-year old male patient was admitted to the emergency department after sustaining a fall from approximately four meters. Full-body CT revealed multiple spine fractures, a blunt thoracic trauma and a multifragmentary subtrochanteric fracture. The femoral fracture was primarily stabilized using an external fixator. The patient was then transferred to the intensive care unit for stabilization

**Fig. 2:**
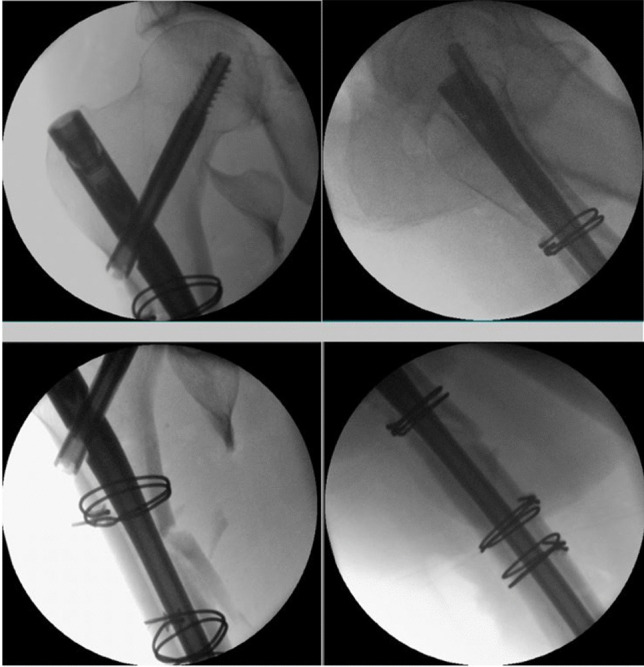
Two days later, definitive treatment with placement of three subtrochanteric cerclages (Depuy/Synthes Cable System) and a long gamma nail was carried out. Reduction of the fracture zone was achieved via an open subvastus approach. Postoperative treatment consisted of early mobilization with limited weight bearing (15 kg)

**Fig. 3:**
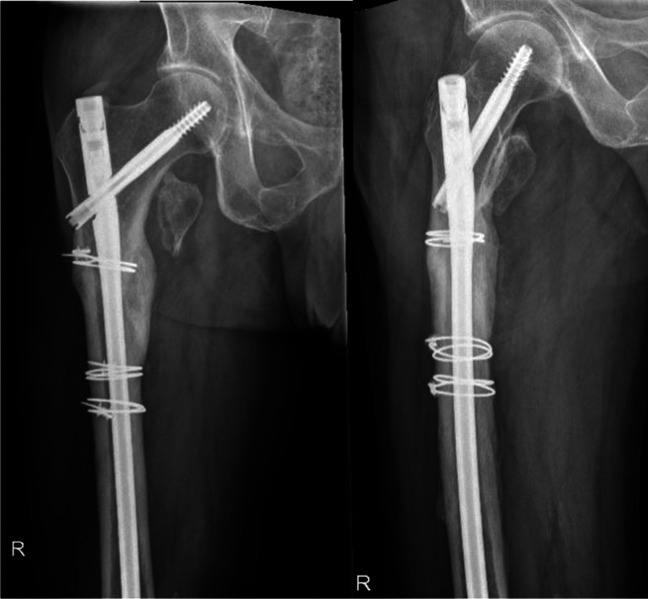
Outpatient follow-up after six weeks showed good results. Consecutively, the patient progressed to full weight bearing. The bone healing process was uneventful. X-ray control one year following surgery demonstrated complete osseous healing of the subtrochanteric fracture and unremarkable position of the implants

Our analyzed patient cohort was mainly male, middle-aged, mostly suffered from high velocity traumas and were frequently diagnosed with multifragmentary subtrochanteric fractures. In the provided figures, the pattern of injury as well as the intraoperative and postoperative X-Rays from a patient of the study cohort are exemplarily illustrated (Figs. 1–3).

One of our major results was, that these fractures were associated with union in 71%, delayed union in 10% (but eventually healed) and nonunion in 19% with the incidence of surgical site infection in 4% and the overall need for revision surgery in 19%.

Multiple studies exist about differences in outcome between open and closed reduction in intramedullary treatment of femoral shaft fractures [19–21]. Although there is common sense, that closed reduction is the preferred technique if an adequate reduction is possible, concrete details about the outcome after open reduction and internal fixation are not well described due to the small sub-cohort in most of the studies [22–24].

### Union/nonunion

Salman et al. performed a systematic review and meta-analysis of 12 studies regarding outcome to compare open to closed reduction methods in intramedullary nailing treatment in femoral shaft fractures [22]. They found a significant better union rate following closed vs. open reduction, with an odds ratio of 2.06 (95% CI, 1.23–3.44). Our incidence of nonunion (19%) is in accordance with those findings, as well as with the results of a retrospective study by Kisan et al., who described a nonunion rate of 14.3% after open vs. 13% after closed reduction [22, 24].

In addition, we found a delayed union rate of 10%, while the review of Kortykowski et al. demonstrated in their review that open reduction was associated with a significantly longer time to union, which was reported by ten studies [25]. In the closed reduction group a time to union was 22 weeks, compared to 24 weeks in the open reduction group [25].

Overall, we noted a union rate of 81% without revision surgery, which is a good result considering that the technique of open reduction should be preferably applied in complex fractures when the attempt of closed reduction failed.

### Infections

Korytkowski et al. performed a meta-analysis of comparative studies and described a lower overall infection rate in the closed reduction cohort ((OR = 0.581; 95% CI, 0.344- 0.981; *p* = 0.042) [25]. Similarly, the meta-analysis of Salman et al. revealed that the incidence of infection following open reduction was significantly higher with rates up to 24% compared to the closed reduction group in the analysed studies [22]. Pooled analysis showed a doubled risk of infection (OR = 1.94; 95% CI, 1.16–3.25, *p* < 0.05) [22]. We found a low number of surgical site infections with a rate of 4%. This may be due to the high medical standard and strict use of perioperative antibiotic prophylaxis, nevertheless, we cannot offer conclusive explanation for this low number.

### Revision surgeries

Altogether, we registered a surgical revision rate of 19%. Salman et al. reported similar findings, but no significant difference in revision rates between both groups (OR = 0.85; 95% CI, 0.53–1.35) [22]. Our rate is based on the avascular femoral head necrosis as well as the nonunions.

### Malunion

We did not observe any malunion. We attribute this to improved intraoperative control for restoring length, axis and rotation with open reduction techniques.

According to the literature malalignment was described to be significantly less with an OR of 0.32 in the open-reduction group in the literature, which shows the strength of this method to achieve a better anatomical reduction compared to closed reduction [22]. Malrotation is one of the most frequent malalignments following intramedullary nailing [26]. Even though not always clinically symptomatic, these malrotations can lead to an increased incidence of osteoarthritis of the hip, knee and ankle [27]. Karaman et al. showed that patients treated with closed reduction and intramedullary nailing with a malrotation of more than 10 degrees, had significantly lower functional scores compared to the cohort without rotational malalignment [28]. Malrotation preventing the achievement of the neutral rotational position bears the highest risk for clinical complaints [29]. This underlines the benefit of open-reduction techniques for precise and anatomical reconstruction in femoral shaft fractures.

In the past, there had been adverse discussions about the use of cerclage wiring which was believed to cause impairment of periostal perfusion and regional bone vascularity. This assumption had been refuted by studies describing no relevant effects of the cerclage wires on periosteal perfusion [30, 31].

In our study we used in 36% of cases two cerclages but had applied up to four cerclages per femur in selected cases of 13% which underscores the strategy of minimizing additional soft tissue trauma by confinement of the number of cerclages.

Regarding the limitations of our study, it is of paramount importance to acknowledge the scope of our dataset derived from a single center. As we focused in this study mainly on the description of the outcome of the patients treated with open reduction and cerclage wiring, our study lacks a CRIF cohort as a control group. As such, we were not able to compare directly a CRIF cohort with the ORIF group in terms of outcome. This constraint inherently limits to draw conclusions/the generalizability of our findings across a broader spectrum of clinical settings and populations. Associated injuries might necessitate different surgical approaches, rehabilitation protocols, and post-operative care, all of which could influence the outcomes observed with cerclage wiring.

Additionally, the diversity of cerclage materials and designs used in clinical practice further complicates the extrapolation of our findings. Different materials may have varying effects on bone vascularity and bone healing outcomes, and the design of the cerclage system (e.g., the number of wires used, their configuration) could also play a critical role.

Since we only included patients with cerclage wiring left in situ, the question if the reduction aid should be removed after implantation of the intramedullary nail or not cannot be addressed. This topic is still a matter of ongoing debate and will need further investigations.

## Conclusion

Although most of femoral shaft fractures are simple, complex fracture pattern represent challenges for the clinical care, as these are usually due to high energy injuries and often associated with critical conditions of the patient [32]. Furthermore, restoring length, axis and rotation can be challenging. Failure in achieving these goals can lead to malunion, nonunion and significant morbidity.

If closed reduction does not lead to sufficient anatomical reduction, the use of open reduction and retention with the help of cerclages specifically for oblique and spiral fractures patterns is, in our opinion, a good option in case of stabilization with an intramedullary nail (Appendix 1). However, the linked risk of morbidity is apparently increased. Apart from this, there is still an ongoing discussion about whether these reduction aids should be removed after implanting the intramedullary nail at the end of the operative procedure.

In line with the current literature our findings suggest that the treatment of choice for femoral shaft fractures is preferably closed reduction and internal fixation with a locking nail. If closed reduction cannot be adequately achieved due to the complex fracture pattern, we suggest the use of ORIF with the assistance of cerclages. As this option is associated with a higher complication rate, an individual risk–benefit evaluation should be performed. Further studies are required to focus on the impact of the number of cerclages as well as their particular localization and the corresponding outcome.

## Data Availability

No datasets were generated or analysed during the current study.

## Appendix

See (Figs. 1, 2, and 3).

See (Tables 1, 2, and 3).

**Table 1:** Overview-patient and fracture characteristics

		*n* = 69
Gender	Male	48 (70%)
	Female	21 (30%)
Age in years		50 ± 21
		(range 16—92)
Trauma mechanism	High velocity	46 (67%)
	Low velocity	23 (33%)
Fracture localization	Unilateral	65 (97%)
	Bilateral	2 (3%)
Fracture type	Pertrochanteric simple	1 (1%)
(*n* = 69)	Pertrochanteric complex	4 (6%)
	Subtrochanteric simple	4 (6%)
	Subtrochanteric complex	24 (35%)
	Shaft simple	8 (12%)
	Shaft complex	13 (19%)
	Combination/Multilevel	15 (22%)
	Open fracture	6 (9%)
Definitive surgery	within first 24 h	39 (57%)
Implant	Gamma3 long nail (Stryker)	51 (74%)
	Expert lateral femoral nail (Synthes)	11 (16%)
	Other intramedullary nail	7 (10%)

**Table 2:** Overview-procedures

		*n* = 69
Time of the day	Day	43 (62%)
	Night	26 (38%)
Level of surgeon	Senior attending	14 (20%)
	Junior attending	44 (64%)
	Resident	11 (16%)
Duration	In minutes	199 ± 77
		(range 75—405)
Surgical approach	Subvastus	57 (83%)
	Transmuscular	8 (12%)
	Unknown	4 (6%)
Degree of reduction	Good	52 (75%)
	Acceptable	13 (19%)
	Poor	4 (6%)
Cerclages (quantity)	4 per femur	9 (13%)
	3	15 (22%)
	2	25 (36%)
	1	20 (29%)
Cerclages (localization)	Above lesser trochanter	12 (18%)
	Below lesser trochanter	68 (98%)

**Table 3:** Overview-outcome

Length of follow-up	In months		36 ± 22
			(range 12—84)
Bone healing	Primary healing		49 (71%)
	Delayed union, but eventually healed		7 (10%)
	Non-union		13 (19%)
		Revised and healed	5 (7%)
		Revised, not healed yet	3 (4%)
		Not yet revised	1 (1%)
		Lost to follow-up after revision	3 (4%)
		Lost to follow-up before revision	1 (1%)
Femoral head necrosis			3 (4%)
Surgical site infection			3 (4%)
Re-operation			13 (19%)
	Re-osteosynthesis		8 (12%)
	Prosthesis		3 (4%)
	Local wound revision		2 (3%)
Hardware removal			21 (30%)
	Surgical site infection after nail removal		1 (5%)
